# TERT and Akt Are Involved in the Par-4-Dependent Apoptosis of Islet *β* Cells in Type 2 Diabetes

**DOI:** 10.1155/2018/7653904

**Published:** 2018-08-14

**Authors:** Chen Liu, Wu QiNan, Lei XiaoTian, Yang MengLiu, Gan XiaGuang, Leng WeiLing, Liang ZiWen, Zhang Ling, Yang GangYi, Chen Bing

**Affiliations:** ^1^Endocrine Department, First Affiliated Hospital of the Third Military Medical University (Army Medical University), Chongqing 400038, China; ^2^Endocrine Department, Second Affiliated Hospital of Chongqing Medical University, Chongqing 400010, China; ^3^Outpatient Department, First Affiliated Hospital of the Third Military Medical University (Army Medical University), Chongqing 400038, China

## Abstract

Islet *β* cell apoptosis plays an important role in type 2 diabetes. We previously reported that Par-4-mediated islet *β* cell apoptosis is induced by high-glucose/fatty acid levels. In the present study, we show that Par-4, which is induced by high-glucose/fatty acid levels, interacts with and inhibits TERT in the cytoplasm and then translocates to the nucleus. Par-4 also inhibited Akt phosphorylation, leading to islet *β* cell apoptosis. We inhibited Par-4 in islet *β* cells under high-glucose/fatty acid conditions and knocked out Par-4 in diabetic mice, which led to the up-regulation of TERT and an improvement in the apoptosis rate. We inhibited Akt phosphorylation in islet *β* cells and diabetic mice, which led to aggressive apoptosis. In addition, the biological film interference technique revealed that Par-4 bound to TERT via its NLS and leucine zipper domains. Our research suggests that Par-4 activation and binding to TERT are key steps required for inducing the apoptosis of islet *β* cells under high-glucose/fatty acid conditions. Inhibiting Akt phosphorylation aggravated apoptosis by activating Par-4 and inhibiting TERT, and Par-4 inhibition may be an attractive target for the treatment of islet *β* cell apoptosis.

## 1. Introduction

Previous studies have shown that *β* cell apoptosis and dysfunction are significantly increased in patients and animals with type 2 diabetes [1–3]. Islet *β* cell apoptosis has been found to be the main cause of islet *β* cell dysfunction and plays an important role in type 2 diabetes in humans [2, 4]. These results suggest that apoptosis is a major cause of type 2 diabetes. Therefore, the mechanism of islet *β* cell apoptosis in type 2 diabetes has attracted substantial attention from diabetes researchers, who believe that hyperglycaemia and hyperlipidaemia in type 2 diabetes can induce endoplasmic reticulum (ER) stress, thereby inducing islet *β* cell apoptosis and dysfunction [4].

Telomerase consists of an RNA template and protein; human telomerase reverse transcriptase (TERT) is the main component of the catalytic telomerase protein subunit responsible for the synthesis function of telomerase [5]. TERT can inhibit apoptosis by activating telomerase. TERT overexpression has an antiapoptotic effect on islet *β* cells, providing a novel target for the treatment of diabetes [6, 7]. However, the antiapoptotic mechanism of TERT is unclear.

Prostate apoptosis response 4 (Par-4) is considered a proapoptotic factor. Previous studies have revealed that Par-4 is involved in various age-related diseases [8]. Par-4 exhibits a nuclear localization sequence (NLS) in its N-terminal region and a leucine zipper domain; the protein can translocate to the nucleus and inhibit Akt to induce tumour cell apoptosis [9, 10]. Par-4 initiates ER stress, which can also increase Par-4 secretion, triggering and intensifying the cell membrane apoptosis pathway. Therefore, ER stress-induced Par-4 secretion can form a vicious cycle, continuously inducing apoptosis. Moreover, Par-4 can also induce apoptosis through the mitochondrial pathway [10]. Although there have been few previous studies on the relationship between Par-4 and diabetes, the fact that ER stress is a common basis of diabetes and cancer indicates that Par-4 may play a role in diabetes. Our previous research revealed that Par-4 activates the transcription level of NF-*κ*B and induces islet *β* cell apoptosis. This process differs from tumour cell apoptosis, in which NF-*κ*B is often down-regulated. Therefore, there may be differences in the mechanism of apoptosis induction by Par-4 between diabetes and tumour cells. We further found that Par-4 can interact with TERT in a yeast two-hybrid system, and other studies have indicated that the interaction between Par-4 and TERT may inhibit TERT in the cytoplasm of nasopharyngeal carcinoma cells, leading to apoptosis. The interaction of Par-4 and TERT differs from typical telomerase-dependent apoptosis regulation, as TERT often serves as the reverse transcriptase, which indicates that this process may represent a novel target in apoptosis regulation [11–15]. In this context, the questions of whether this process is consistent between tumour cells and the apoptosis of islet *β* cells in diabetes, whether there is any association between the interaction of Par-4 with TERT and Par-4 nuclear translocation in islet *β* cell apoptosis, and if any relationship exists between Par-4 and Akt in the apoptosis of islet *β* cells remain to be investigated.

Therefore, we herein report for the first time a novel role of Par-4 interaction with TERT, followed by nuclear translocation to induce islet *β* cell apoptosis, and we reveal the relationship between Par-4 and Akt signalling in the apoptosis of islet *β* cells in type 2 diabetes. We show that Par-4 has an inhibitory effect on TERT and Akt to induce apoptosis of islet *β* cells in the pathology of diabetes. Small interfering RNA- (siRNA-) mediated inhibition of Par-4 increases the expression of TERT and p-Akt and has a relief effect on islet *β* cell apoptosis. We also demonstrate that TERT can bind to Par-4 directly. Our findings suggest that the Par-4/TERT-Akt pathway plays an important role in the apoptosis of islet *β* cells in type 2 diabetes.

## 2. Materials and Methods

### 2.1. Patient Recruitment and Identification

There were 60 patient samples examined: thirty newly diagnosed type 2 diabetes patients and 30 healthy people were recruited for the study; there were 14 male patients and 16 female patients in the control group, and 15 male patients and 15 female patients in the diabetes group; the average age is 55 ± 11 years in the control group and 54 ± 8 years in the diabetes group. The study protocol was approved by the Southwest Hospital of the Third Military Medical University Institutional Review Board and conformed to the standards of the Declaration of Helsinki. Only clinical data and fasting blood samples were taken and analysed. The inclusion criteria for the type 2 diabetes group were described previously [16].

### 2.2. Inhibition of Par-4

siRNA was designed, and siRNA experiments were performed by the Shanghai China GenePharma Co. Ltd. as described previously [15].

### 2.3. Cell Culture and Reagents

The mouse insulinoma cell line NIT-1 was kindly provided by Dr. Chen Li Qing (Third Military Medical University, China). NIT-1 cells were grown in Dulbecco's Modified Eagle's medium (DMEM, HyClone, with a glucose concentration of 5 mmol/L) supplemented with 10% foetal bovine serum (HyClone) and 1% penicillin/streptomycin. Palmitic acid (Sigma) was dissolved at a concentration of 100 nmol/L in DMEM containing 0.5% bovine serum albumin (BSA) (Sigma). The cells were treated as described previously [15]. We used the intervention method of reference 14, but the intervention time has been adjusted to 48 h based on our results of the experiment.

### 2.4. Animal Experiments

All animal experiments were performed in accordance with the approval of the Institutional Animal Care and Use Committee. C57BL/6J male mice were obtained from the animal centre of the Third Military Medical University, Chongqing. Par-4-knockout C57BL/6J male mice were purchased from Cyagen Biosciences Inc. The process of Par-4 KO mice: The mPawr gene (GenBank accession number: NM_054056.2; Ensembl: ENSMUSG00000035873) is located on mouse chromosome 10. Exon 2 was selected as the target site. TALEN mRNA generated by in vitro transcription was then injected into fertilized eggs for KO mouse productions. The founders were genotyped by PCR followed by DNA sequencing analysis. The positive founders were breeding to the next generation which was genotyped by PCR and DNA sequencing analysis. (see Supplementary Materials). Type 2 diabetes was induced in the mice as described previously [14]. Eight C57BL/6J mice were randomly assigned to the N group, and 8 Par-4-knockout C57BL/6J mice were randomly assigned to the N-Par-4 group. These mice were fed a normal chow diet for 12 weeks. Eight C57BL/6J mice with type 2 diabetes were randomly assigned to the D group, and 8 Par-4-knockout C57BL/6J mice with type 2 diabetes were randomly assigned to the D-Par-4 group. These mice were fed a high-fat diet for 12 weeks. The weight and blood glucose level of each animal were measured daily. At the end of the experiment, the pancreas was removed and stored at −80°C.

### 2.5. Immunohistochemistry

The experiment was performed as described previously [15, 17]. Deparaffinized sections were incubated with a Par-4 (dilution 1 : 200; Santa Cruz Biotechnology, USA) or TERT (dilution 1 : 200; Santa Cruz Biotechnology, USA) primary antibody overnight at 4°C.

### 2.6. Western Blotting

The experiment was performed as described previously [15]. Membranes were incubated with Par-4 (1 : 400, Santa Cruz Biotechnology), TERT (1 : 300, Santa Cruz Biotechnology), Akt (1 : 500, Santa Cruz Biotechnology, USA), and p-Akt (1 : 500, Santa Cruz Biotechnology, USA) antibodies at 4°C overnight. Protein expression was detected 3 times for each sample.

### 2.7. Immunoprecipitation

To observe the interaction between Par-4 and TERT, cells were lysed in a RIPA buffer with a protease inhibitor. A rabbit anti-TERT (1 : 400, Santa Cruz Biotechnology) or anti-Par-4 (1 : 400, Santa Cruz Biotechnology) antibody was incubated with the cell lysate for 6 h at 4°C, and the resulting complexes were then precipitated with protein A/G-Sepharose (sc-2003, Santa Cruz Biotechnology) overnight at 4°C. Next, the precipitates were washed 5 times with PBS at 0°C, separated via SDS-PAGE and probed with a rabbit anti-TERT (1 : 400, Santa Cruz Biotechnology) or rabbit anti-Par-4 (1 : 400, Santa Cruz Biotechnology) antibody in western blotting.

### 2.8. Apoptosis Detection

TUNEL staining to detect the apoptosis rate and corresponding calculations were performed as previously described [15, 17].

### 2.9. Measurement of Insulin after Glucose Stimulation

Cells were plated in 48-well plates at a density of 1 × 10^5^ cells/well. After 16 h, the medium was removed and the cells were washed once and then incubated for 1 h in a glucose-free Krebs-Ringer bicarbonate (KRB) buffer (115 mM NaCl, 4.7 mM KCl, 1.2 mM MgSO_4_·7H_2_O, 1.2 mM KH_2_PO_4_, 20 mM NaHCO_3_, 16 mM HEPES, 2.56 mM CaCl_2_, and 0.2% BSA). The cells were next treated in a KRB buffer with a high-glucose concentration (25 mM) for 1 h. Thereafter, the medium was collected and stored at −20°C for subsequent ELISA analysis (EZRMI-13K, Millipore) to determine insulin secretion. To determine the insulin content, cells were lysed with 0.1% Triton X-100 and insulin contents and secretion were normalized to the total protein content using the bicinchoninic acid (BCA) method. Changes in insulin secretion and content were then calculated with reference to the results obtained from the control group [15].

### 2.10. Measurement of Par-4 Secretion

Par-4 secretion was detected with a Par-4 ELISA kit (MBL). The experiment was described previously [15].

### 2.11. Clinical Sample Analysis

The age, height, weight, BMI, BP, and disease course of the patients providing the samples were obtained. Fasting plasma glucose, HbA1c, fasting insulin, HOMA-*β*, total cholesterol (TC), triglyceride (TG), low-density lipoprotein cholesterol (LDL-C), and high-density lipoprotein (HDL-C) were tested by a certified laboratory. Par-4 was measured with a commercially available ELISA kit (Fujirebio, Tokyo, Japan). TERT was detected via ELISA (R&D Systems Inc., USA) following the manufacturer's instructions [16].

### 2.12. Statistical Analysis

Statistical software SPSS 19.0 was used for these analyses. The data are presented as the mean ± standard deviation (SD). The differences between groups were evaluated using ANOVA for multiple comparisons. Correlations between variables were identified through Spearman's correlation (rs) and were used to correct for the effects of independent factors.

## 3. Results

### 3.1. Par-4 and TERT Are Associated with Islet *β* Cell Dysfunction in Type 2 Diabetes Patients

**Figure 1:**
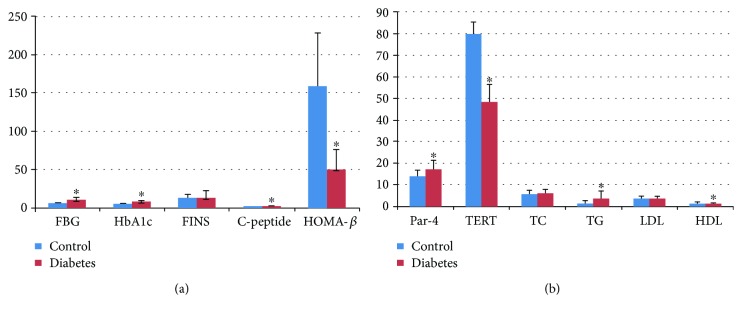
Parameters in type 2 diabetes patients and healthy subjects. ^∗^Compared with the control group, *P* < 0.05.

Whether Par-4 and TERT are involved in islet beta cell dysfunction in the course of type 2 diabetes has not previously been reported. Hence, we detected the concentrations of Par-4 and TERT in newly diagnosed type 2 diabetes patients. There were significant differences in fasting blood glucose (FBG), HbA1c, C-peptide, HOMA-*β*, Par-4, TERT, TG, and HDL-C (*P* < 0.05) between the type 2 diabetes group and the control group. The serum level of Par-4 was negatively correlated with the HOMA-*β* index (*r* = −0.365, *P* = 0.004) and C-peptide (*r* = −0.337, *P* = 0.008), whereas it was not significantly correlated with FBG, FINS, or blood lipid levels. Serum Par-4 and TERT levels (*r* = −0.362, *P* = 0.004) were negatively correlated. For detailed results, see Figure 1 and Supplementary Tables 1–3.

Although these results did not directly reflect the *β* cell concentrations of Par-4 and TERT, they indicated that Par-4 and TERT may show some relationship with islet *β* cell dysfunction in type 2 diabetes. There was no correlation between Par-4 and FBG, insulin, or blood lipid levels; therefore, Par-4 is not solely responsible for islet *β* cell dysfunction. Because we could not collect pancreatic tissue from the patients with diabetes, the subsequent in vitro research was intended to explore the mechanism of Par-4 in apoptosis and islet *β* cell function.

### 3.2. Par-4 Expression Is Positively Associated with Apoptosis and Negatively Associated with TERT

**Figure 2:**
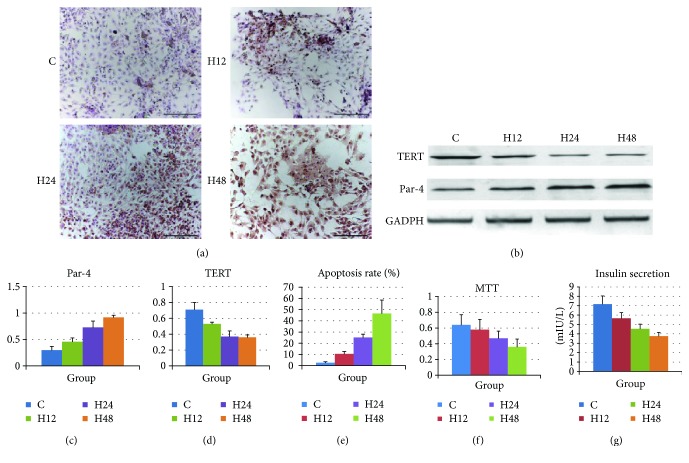
Diabetes up-regulates Par-4, down-regulates TERT, induces islet *β* cell apoptosis, and decreases the survival rate and glucose-induced insulin secretion. (a) Effect of high-glucose/palmitate treatment for 12 h, 24 h, and 48 h on NIT-1 cells. The control group was treated with DMSO for 48 h. Cells were exposed simultaneously to high glucose (25.5 mmol/L) and palmitate (100 nmol/L) for 12 h, 24 h, or 48 h and then harvested, and cell lysates were prepared. The total cellular lysates were subjected to western blot analysis using antibodies against Par-4 and TERT. (b) Apoptosis was detected in each group of cells via TUNEL staining. Expression of Par-4 (c) and TERT (d), apoptosis rates (e), cell survival rates determined in MTT assays (f), and glucose-stimulated insulin secretion (g) in each group. ^∗^Compared with the C group, *P* < 0.05. ^#^Compared with the H24 group, *P* < 0.05.

There are no available reports on the roles of Par-4 and TERT in islet *β* cell apoptosis in type 2 diabetes. Therefore, we further investigated the association between Par-4 and TERT in islet *β* cell apoptosis induced by high-glucose/palmitate levels, which mimics type 2 diabetes. The cells were divided into 4 groups: C (control, grown under normal culture conditions for 48 h), H12 (high-glucose/palmitate treatment for 12 h), H24 (high-glucose/palmitate treatment for 24 h), and H48 (high-glucose/palmitate treatment for 48 h). Par-4 and TERT expression in each group was detected by western blotting; apoptosis was evaluated by TUNEL staining; the cell survival rate was determined using MTT assays; and glucose-stimulated insulin secretion was calculated based on ELISA results. As the treatment time increased, Par-4 expression and the apoptosis rate increased significantly in cells treated with high glucose/palmitate compared with that in control cells and cells treated for shorter periods of time (*P* < 0.05). Furthermore, TERT expression, the survival rate, and insulin secretion were significantly decreased in cells treated with high glucose/palmitate compared with those in control cells and those treated for shorter periods of time (*P* < 0.05) (Figures 2(b)–2(f)).

These data suggested that Par-4 expression is positively associated with high-glucose/palmitate treatment and the apoptosis rate and negatively associated with TERT expression, the survival rate, and insulin secretion and, further, that these effects are time dependent. Therefore, 48 h was selected as the optimal treatment time.

### 3.3. High-Glucose/Palmitate Treatment Induces Apoptosis of Islet *β* Cells via Up-Regulation of Par-4 Expression and Down-Regulation of TERT Expression

**Figure 3:**
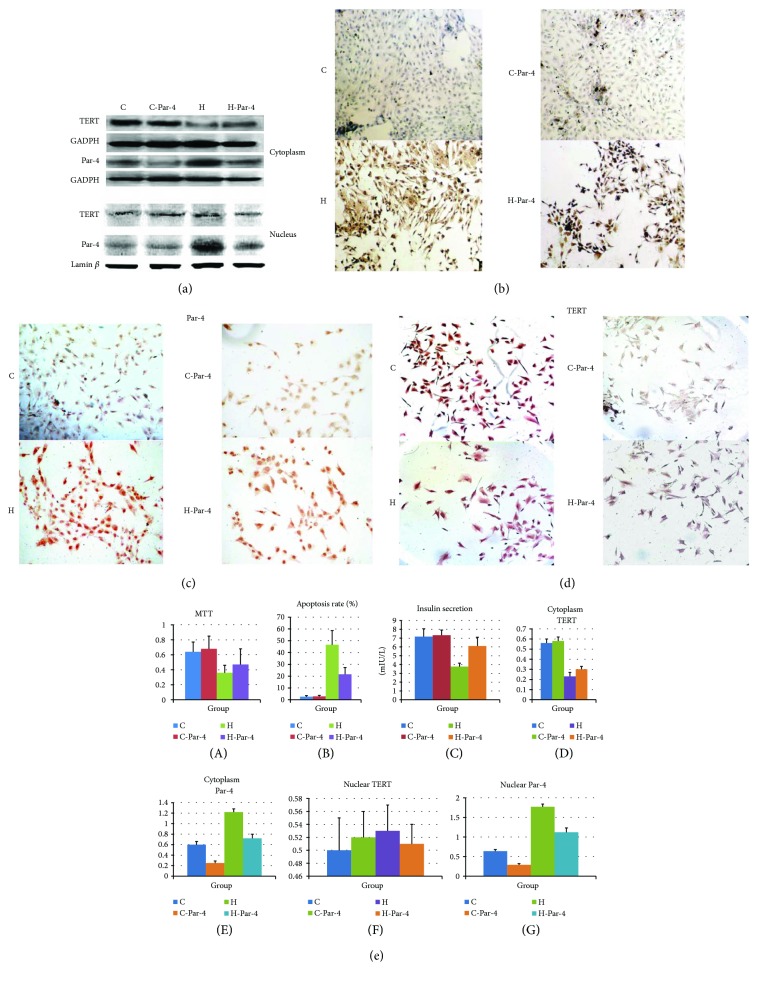
Subcellular localization of Par-4 and TERT in diabetes. (a) Western blot analysis of Par-4 and TERT expression in cytoplasmic and nuclear extracts in each group. (b) Apoptosis was detected via TUNEL staining in each group. (c) Immunocytochemistry analysis of Par-4 expression in each group. (d) Immunocytochemistry analysis of TERT expression in each group. (e) Expression of Par-4 and TERT, apoptosis rates, cell survival rates determined with MTT assays, and glucose-stimulated insulin secretion in each group. ^∗^Compared with the C group, *P* < 0.05. ^#^Compared with the H group, *P* < 0.05.

Because Par-4 is negatively associated with TERT and positively associated with apoptosis, we next sought to determine whether Par-4 signalling affects TERT levels and apoptosis. NIT-1 cells were divided into a control group (C group), a high-glucose/palmitate treatment group (H group), a Par-4 inhibition group (C-Par-4 group), and a high-glucose/palmitate intervention + Par-4 inhibition group (H-Par-4 group), and the treatments were administered as described previously [14]. Western blot analysis showed that Par-4 and TERT protein expression in cytoplasmic extracts was not significantly different in the C-Par-4 group compared with that in the C group (*P* > 0.05). In the H and H-Par-4 groups, Par-4 expression was significantly higher than that in the C group (*P* < 0.05), and TERT expression was significantly lower than that in the C group (*P* < 0.05). Compared with that in the H group, Par-4 expression in the H-Par-4 group was significantly decreased (*P* < 0.05) and TERT expression was significantly increased (*P* < 0.05) (Figures 3(a) and 3(e)).

We performed an MTT assay and TUNEL staining in each group, and the glucose-stimulated insulin secretion assay was performed after 1 h of high-glucose (25 mM) stimulation in all groups (4.9 measurement of insulin after glucose stimulation). Compared with the C group, the C-Par-4 group showed similar levels of cell survival, apoptosis, and insulin secretion (*P* > 0.05), which indicated that Par-4 inhibition in normal cells may not alter cell survival, apoptosis, or insulin secretion. In the H and H-Par-4 groups, the apoptosis rate was significantly higher than that in the C group (*P* < 0.05), and the cell survival rate and insulin secretion were significantly lower than those in the C group (*P* < 0.05). Compared with the H group, the H-Par-4 group exhibited a decreased apoptosis rate (*P* < 0.05) and an increased survival rate and insulin secretion (*P* < 0.05) (Figures 3(b) and 3(e)).

We also analysed nuclear protein expression. Compared with the C group, the C-Par-4 group did not exhibit any difference in Par-4 and TERT protein expression (*P* > 0.05), indicating that inhibition of Par-4 in normal cells may not alter the nuclear expression of Par-4 and TERT. Par-4 expression was significantly higher in the H and H-Par-4 groups than that in the C group (*P* < 0.05), but TERT expression was not significantly different in these groups compared with that in the C group (*P* > 0.05). Compared with the H group, the H-Par-4 group showed decreased Par-4 expression (*P* < 0.05), but no significant difference in TERT expression (*P* > 0.05) (Figures 3(a) and 3(e)).

These results suggested that high-glucose/fatty acid treatment can increase cytoplasmic Par-4 levels and the apoptosis rate and decrease cytoplasmic TERT levels, cell survival, and the insulin secretion ability, while knockdown of Par-4 can increase cytoplasmic TERT levels, cell survival, and the insulin secretion ability under high-glucose/fatty acid conditions.

### 3.4. Par-4 Interacts with TERT, and Inhibition of TERT in the Cytoplasm, as Observed in Diabetes, Triggers Par-4 Translocation to the Nucleus

**Figure 4:**
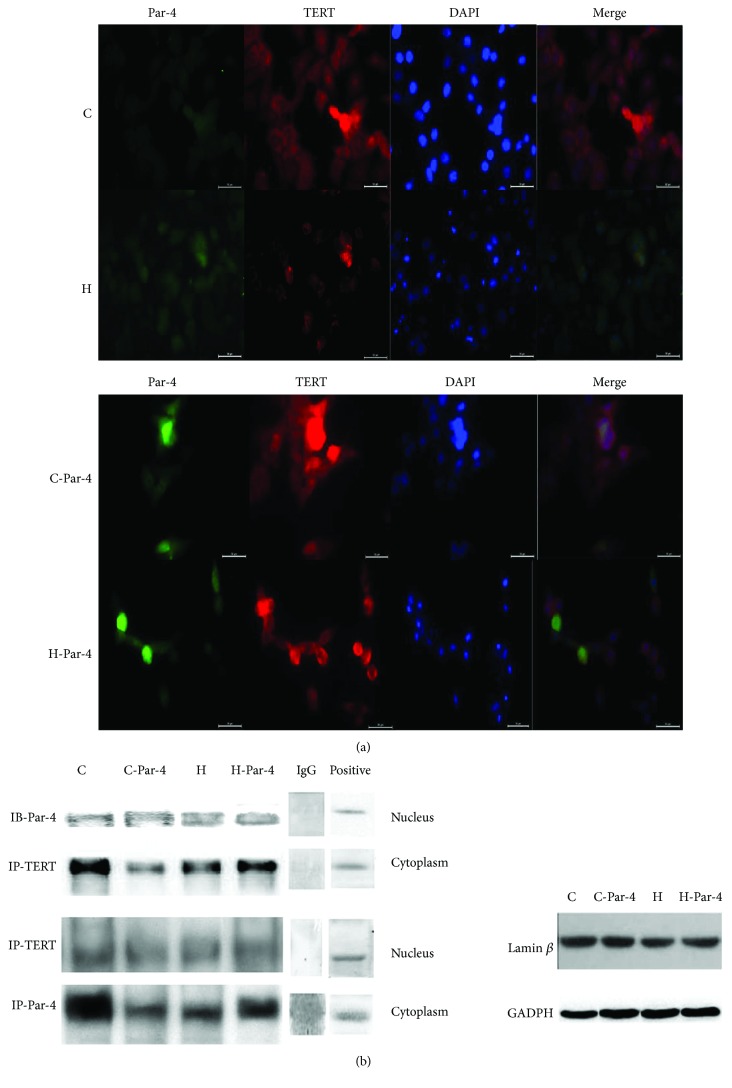
Subcellular localization of Par-4 and TERT in NIT cells. (a) Immunofluorescence of Par-4 and TERT expression in each group. (b) Immunoprecipitation of Par-4 and TERT expressed in each group.

Some studies have indicated that Par-4 interacts with TERT to regulate the cell apoptosis, but whether the interaction between Par-4 and TERT is involved in islet *β* cell apoptosis in diabetes remains unknown. Therefore, we next detected the interaction between Par-4 and TERT via immunoprecipitation and immunofluorescence staining. Compared with the C group, the H group showed significantly increased cytoplasmic and nuclear Par-4 expression, but clearly decreased TERT expression in the cytoplasm, while nuclear TERT expression was not affected. In the H-Par-4 group, both cytoplasmic and nuclear Par-4 expressions decreased, while cytoplasmic TERT expression increased, and nuclear TERT staining was not affected. We observed both red fluorescence (TERT staining) and green fluorescence (Par-4 staining). Merged images showed that in the C and C-Par-4 groups, TERT was primarily localized in the cytoplasm, as the nuclei were either blue or purple. In the H group, green fluorescence (Par-4 staining) was stronger than that in the C group, no red fluorescence was visible in the cytoplasm, and nuclear staining was cyan, mainly because of stronger green fluorescence in the nucleus which added to the blue staining of DAPI. In the H-Par-4 group, green fluorescence in the cytoplasm was decreased compared with the H group, while increases in red fluorescence and blue DAPI fluorescence were observed (Figure 4(a)).

Compared with the H group, the Par-4-TERT interaction was significantly increased in the H-Par-4 group (*P* < 0.05). The Par-4-TERT interaction was significantly weaker (*P* < 0.05) in the C group than in the H group and was increased significantly (*P* < 0.05) in the H-Par-4 group compared with that in the C group. These findings, along with the previous results, indicate that the main site of interaction between TERT and Par-4 is the cytoplasm. Compared with the C group, Par-4 expression and the Par-4-TERT interaction were increased in the H group, and TERT was inhibited in the cytoplasm by binding with the increased number of Par-4 molecules. Par-4-TERT binding was also increased, although the nuclear expression of Par-4 increased as well. Inhibiting Par-4 expression could reduce these events and effects (Figure 4(b)).

These results suggested that Par-4 and TERT interact in the cytoplasm and inhibit each other's expression. High-glucose/palmitate treatment induced Par-4 expression, inhibited TERT, and increased apoptosis. In contrast, reducing Par-4 expression under high-glucose/palmitate conditions increased TERT expression, thereby reducing apoptosis. TERT expression in the cytoplasm may be a cellular self-protective mechanism, as TERT interacts with Par-4 and inhibits its expression. The detailed mechanism of this interaction was explored in the following experiment.

### 3.5. Diabetes Activates Par-4 and Inhibits P-Akt Induction to Induce Islet *β* Cell Apoptosis

**Figure 5:**
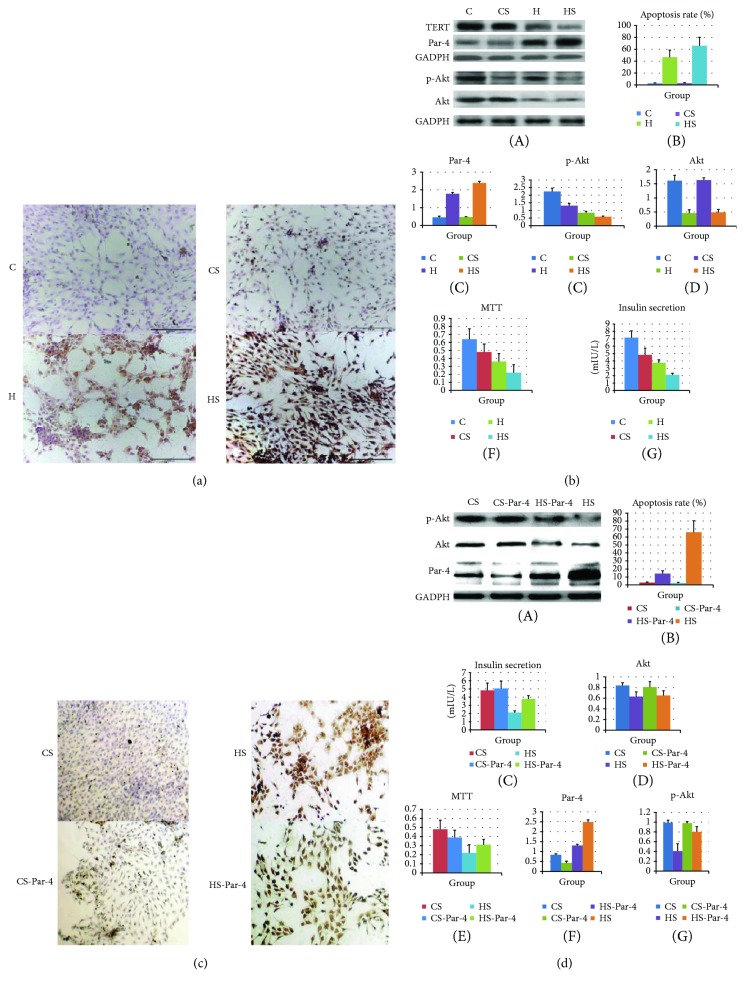
Diabetes activates Par-4 and inhibits p-Akt, inducing NIT-1 cell apoptosis. (a) Apoptosis was detected via TUNEL staining in the C, CS, H, and HS groups. (b) Western blot analysis of Par-4, Akt, and p-Akt expression; apoptosis rates; cell survival rates determined with MTT assays; and glucose-stimulated insulin secretion in the C, CS, H, and HS groups. (c) Apoptosis was detected via TUNEL staining in the CS, HS, CS-Par-4, and HS-Par-4 groups. (d) Western blot analysis of Par-4, Akt and p-Akt expression, apoptosis rates, cell survival rates determined with MTT assays, and glucose-stimulated insulin secretion in the CS, HS, CS-Par-4, and HS-Par-4 groups. ^∗^Compared with the C group, *P* < 0.05. ^#^Compared with the H group, *P* < 0.05. ^a^Compared with the CS group, *P* < 0.05. ^b^Compared with the HS group, *P* < 0.05.

**Figure 6:**
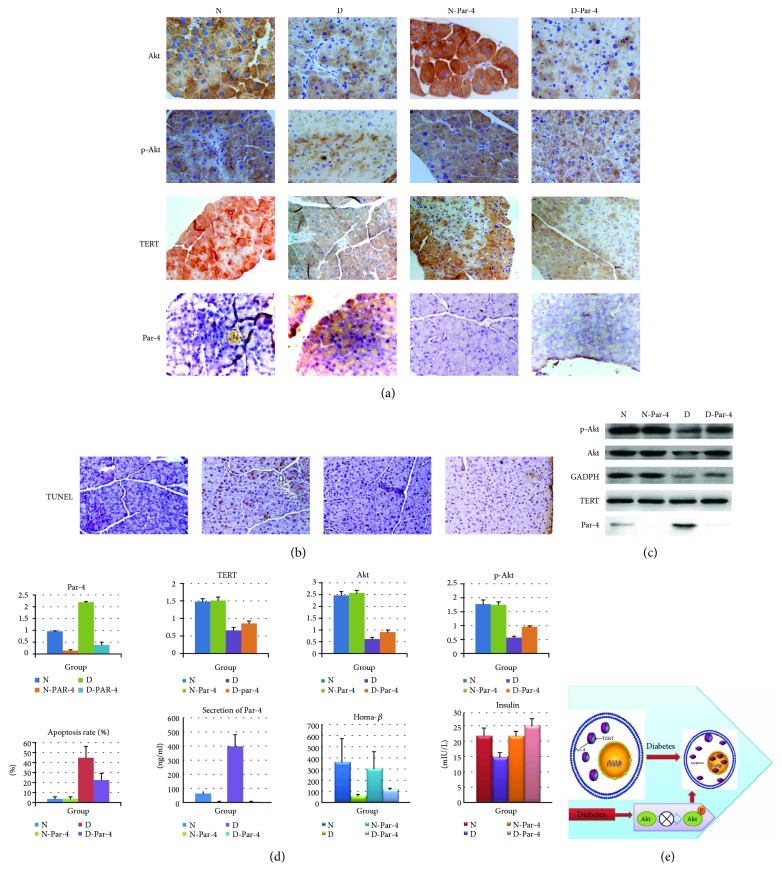
Diabetes activates Par-4 and inhibits TERT and p-Akt, inducing islet *β* cell apoptosis. (a) Immunocytochemistry analysis of Par-4 and TERT expression in cytoplasmic and nuclear extracts in each group. (b) Apoptosis was detected via TUNEL staining in each group. (c) Western blots of Par-4, TERT, Akt, and p-Akt expression in each group. (d) Expression of Par-4 (A), TERT (B), Akt (C), and p-Akt (D), apoptosis rates (E), secretion of Par-4 (F), the HOMA- *β* index (G), and glucose-stimulated insulin secretion (H) in each group. (e) Signal transduction via the Par-4/TERT-Akt pathway to induce islet *β* cell apoptosis in diabetes. ^∗^Compared with the N group, *P* < 0.05. ^#^Compared with the D group, *P* < 0.05.

Akt is an important signalling factor in cell apoptosis and is induced by Par-4. However, there are no available reports on the relationship between Par-4 and p-Akt in the apoptosis of islet *β* cells in diabetes. We used RNAi to inhibit Par-4 and employed the Akt inhibitor SH5 to inhibit Akt signalling. Thereafter, we detected the apoptosis rate, survival rate, insulin secretion, and the expression of Par-4 and p-Akt in each group. NIT-1 cells were divided into a control group (C group), a high-glucose/palmitate treatment group (H group), an SH5 inhibition group (CS group), and a high-glucose/palmitate intervention + SH5 inhibition group (HS group); each group was treated with 20 *μ*mol/L of SH5. Compared with the C group, the CS group showed decreased Akt and p-Akt protein expression but no significant change in the apoptosis rate, cell survival, or glucose-stimulated insulin secretion (*P* > 0.05), suggesting that inhibiting Akt in the C group does not affect the apoptosis or survival rate. However, in the H and HS groups, Akt and p-Akt protein expression levels, insulin secretion, and cell survival were significantly lower than those in the C group (*P* < 0.05), and the apoptosis rate was significantly higher than that in the C group (*P* < 0.05) (Figures 5(a) and 5(b), A–G). Compared with the H group, the HS group showed decreases in Akt and p-Akt protein expression levels, insulin secretion, and cell survival (*P* < 0.05) and a significantly higher apoptosis rate (*P* < 0.05) (Figures 6(a) and 6(b), A–G). We also used siRNA to down-regulate Par-4 in the H and HS groups (H-Par-4 and HS-Par-4). Compared with the H and HS groups, the H-Par-4 and HS-Par-4 groups showed increases in Akt and p-Akt protein expression levels, insulin secretion, and cell survival (*P* < 0.05) and a significantly lower apoptosis rate (Figures 5(c) and 5(d), A–G).

These findings suggested that high-glucose/fatty acid treatment can lead to increases in Par-4 expression and secretion and decreases in Akt and p-Akt expression, survival rates, and insulin secretion, resulting in increased NIT-1 cell apoptosis. Par-4 inhibition can alleviate these effects, whereas inhibiting p-Akt aggravates them. Thus, the Par-4-dependent induction of NIT-1 cell apoptosis mainly occurs via the Akt signalling pathway, suggesting that Par-4 may play an important role in high-glucose/fatty acid-induced *β* cell apoptosis and changes in the insulin secretion ability via the Akt signalling pathway.

In the next experiment, we validated the results of the previous experiment in an animal model. We constructed a Par-4-knockout mouse model (Cyagen Biosciences Inc.) and a STZ (streptozotocin) + high-fat diet-induced type 2 diabetic mouse model. The mice were divided into a normal group (N group), a type 2 diabetes group (D group), a normal Par-4-knockout group (N-Par-4 group), and a type 2 diabetes and Par-4-knockout group (D-Par-4 group); the treatments were administered as described previously [17]. Par-4, TERT, Akt and p-Akt levels, insulin secretion, and Par-4 secretion were detected in all groups. The secretory ability of islet cells was evaluated using the HOMA-*β* index, and we detected islet *β* cell apoptosis through TUNEL staining. There were no differences in TERT, Par-4, Akt, or p-Akt protein levels; apoptosis; or insulin or Par-4 secretion (*P* > 0.05) (Figures 6(a), 6(c), and 6(d), A–H). In the D groups, Par-4 expression and secretion were significantly higher than those in the N group (*P* < 0.05) (Figures 6(c) and 6(d), A, F), while TERT, Akt, p-Akt, and insulin secretion were significantly lower than those in the N group (*P* < 0.05) (Figures 6(c) and 6(d), B–H), and apoptosis increased significantly (*P* < 0.05) (Figures 6(b) and 6(d), E). Compared with the D group, TERT, Akt, p-Akt, and insulin secretion were increased (*P* < 0.05) (Figures 6(c) and 6(d), B–H) and apoptosis was decreased in the D-Par-4 group (*P* < 0.05) (Figures 6(b) and 6(d), E).

These results indicated that type 2 diabetes can induce the expression and secretion of Par-4 and reduce the expression of TERT, Akt, and p-Akt, leading to apoptosis and decreased insulin secretion. Inhibiting Par-4 expression can increase the levels of TERT, Akt, and p-Akt; reduce apoptosis; and improve the dysfunction of insulin secretion (Figure 6(e)).

**Figure 7:**
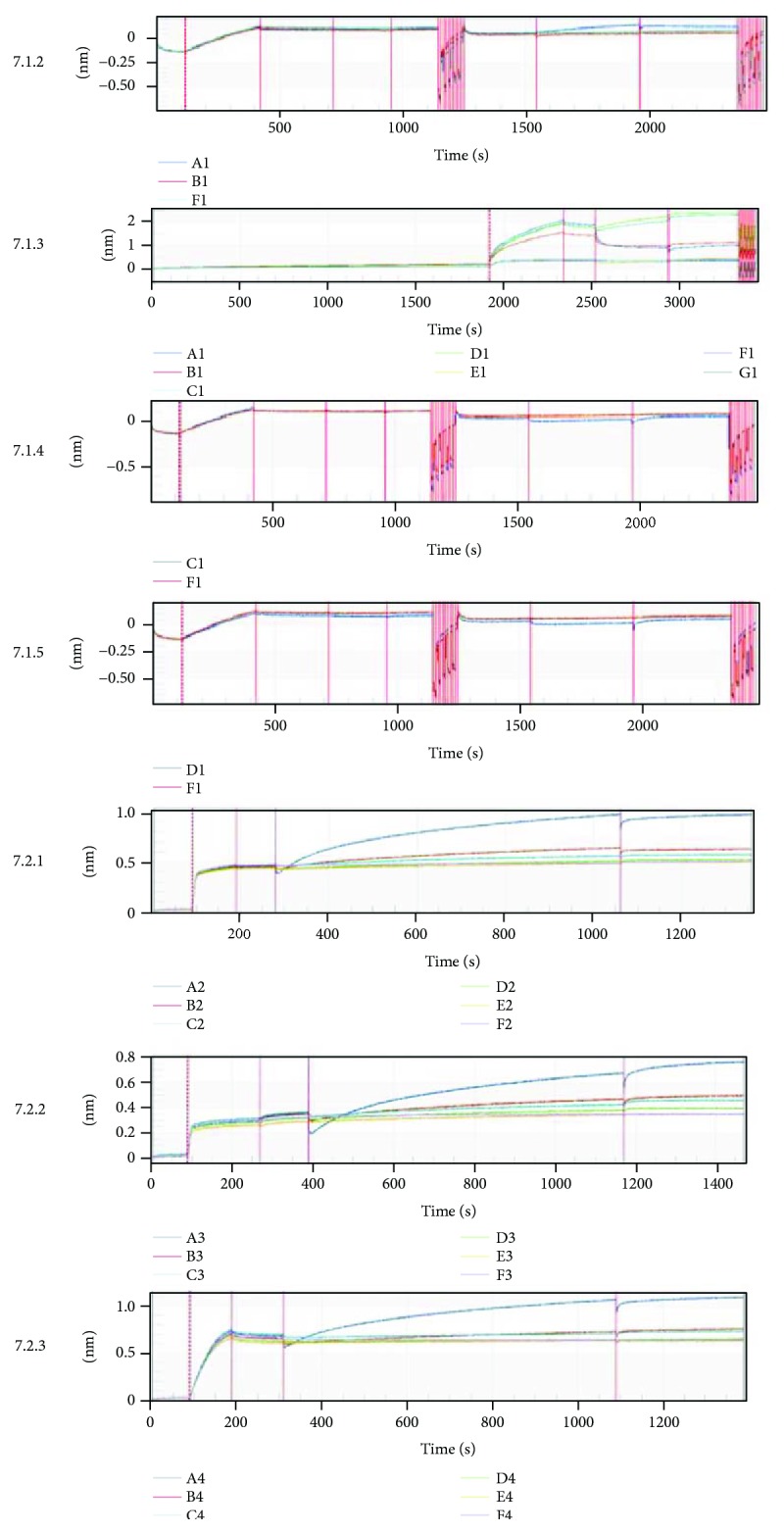
Par-4 binds directly to TERT. (7.1.1) Interaction between Par-4 and TERT, ligand: Par-4 protein, analyte: TERT-1: TERT (1–183); TERT-2: TERT (170–546); TERT-3: TERT (528–924); and TERT-4: TERT (915–1182). (7.1.2) Interaction between Par-4 and TERT-1, A1: TERT-1; E1: FAP-tag; F1: negative control. (7.1.3) Interaction between Par-4 and TERT-2, A1–E1: different concentrations of TERT-2; F1: FAP-tag; G1: negative control. (7.1.4) Interaction between Par-4 and TERT-3, A1: TERT-3; E1: FAP-tag; F1: negative control. (7.1.5) Interaction between Par-4 and TERT-4, A1: TERT-4; E1: FAP-tag; F1: negative control. (7.2) Interaction between Par-4 and TERT-2 (tag free), ligand: Par-4-1: Par-4 (1–160); Par-4-2: Par-4 (161–340); and Par-4-3: full-length Par-4, analyte: TERT (170–546 tag free): TERT-2 (tag free). (7.2.1) Interaction between Par-4-1 and TERT-2 (tag free), A2–D2: different concentrations of Par-4-1; E2: FAP-tag; F2: negative control. (7.2.3) Interaction between Par-4-2 and TERT-2 (tag free), A3–D3: different concentrations of Par-4-2; E2: FAP-tag; F2: negative control. (7.2.4) Interaction between full-length Par-4 and TERT-2 (tag free), A4–D4: different concentrations of Par-4-2; E2: FAP-tag; F2: negative control.

We next sought to determine whether the interaction between Par-4 and TERT occurs directly or indirectly. We constructed Par-4 and TERT missense mutants (TERT-1-4 and Par-4-1-3) and used biological film interference technology to detect interactions between these missense mutants. Only TERT (170–546) could bind to Par-4, while Par-4 (1–160) (Figure 7, 7.1.2–7.1.5 and Supplementary Table 9), Par-4 (161–340), and full-length Par-4 all bound to TERT (170–546) (Figure 7, 7.2.2–7.1.4 and Supplementary Table 10) in vitro. These results reveal a direct interaction between Par-4 and TERT. According to the above results, TERT and Par-4 interact with each other and negatively regulate apoptosis in islet *β* cells.

## 4. Discussion

Islet *β* cell apoptosis is still the main pathological basis of type 2 diabetes [4, 18]. Many studies have indicated that diabetes can induce ER stress and mitochondrial dysfunction, leading to abnormal telomere-telomerase functioning, thereby promoting apoptosis signalling and decreasing the number and function of islet *β* cells [5]. But the specific process is unclear.

TERT is a key factor in telomerase activation due to its role as a reverse transcriptase. TERT mediates DNA synthesis of the template of the telomerase RNA gene to increase the length of telomeres, which is regulated by telomerase. This pathway is referred to as the typical telomere-telomerase system pathway [19, 20]. The accumulation of oxidative stress reduces the self-repair ability of telomeres, leading to telomere attrition, apoptosis, and dysfunction; however, it is not known whether this is the only way to induce cell apoptosis [5]. Many studies have shown that TERT can interact with other signalling factors to relieve its antiapoptotic effect, which is independent of the typical telomere-telomerase system pathway. For example, Cao et al. inhibited TERT expression in human breast cancer cells, resulting in nontelomerase activity-dependent apoptosis, confirming that TERT expression can promote cell survival and proliferation via a telomerase-independent pathway [20]. Dudognon et al. found that exogenous expression of TERT protected cells from TNF- or TRAIL-induced cell apoptosis, and this function did not depend on telomerase activity or telomere maintenance mechanisms [21]. Rahman et al. increased the expression of TERT in BL41 cells, resulting in an increased survival rate of the cells [22]. Furthermore, in HCT116 cells, increased expression of TERT can inhibit P53-dependent cell death [23]. It has been suggested that TERT exerts an antiapoptotic effect that is independent of the telomere-telomerase system, but the specific mechanism is unclear. In previous studies, we and other researchers have found that TERT can interact with Par-4 and inhibits Par-4-induced apoptosis but has no obvious effect on telomerase activity. These findings indicate that Par-4 may be a candidate for relieving the antiapoptotic effect of TERT, which is independent of the typical telomere-telomerase system pathway; however, there has been no further research on this specific process. Moreover, TERT is a potential target involved in the inhibition of islet *β* cell apoptosis, and overexpression of TERT can lead to antiapoptotic effects in islet *β* cells [10–13]. Hence, the question of whether there is any association with the interaction between TERT and Par-4 arises. The mechanism whereby TERT protects islet *β* cells from apoptosis is also in need of further investigation.

Par-4 is a proapoptotic factor that can activate the cell membrane apoptosis pathway and, thus, the caspase cascade programme [24, 25]. We previously found that diabetes increases Par-4 secretion and expression, activates the ER stress-cell membrane and mitochondrial pathways, and mediates the translocation of Par-4 to the nucleus to activate NF-*κ*B transcription and induce apoptosis of islet *β* cells, but the process of Par-4 translocation to the nucleus is unclear [15]. Some studies have found that Par-4 can interact with TERT in the cytoplasm, preventing Par-4 from translocating to the nucleus in tumour cells. Based on the above findings, TERT may interact with Par-4 in the cytoplasm, thereby inhibiting Par-4 translocation to the nucleus to regulate islet *β* cell apoptosis in type 2 diabetes [10–13]. Furthermore, TERT expression is decreased in diabetes, leading to the question of whether this decreased TERT level meets the requirement for Par-4 inhibition and results in the translocation of Par-4 to the nucleus to induce apoptosis. In addition, the leucine zipper region of Par-4 is the key site for Akt binding to Par-4, and this region is also important in the regulation of Par-4-mediated apoptosis [10]. Par-4 can inhibit the Akt signalling pathway to promote apoptosis, and our previous studies have shown that ER stress promotes the expression and secretion of Par-4, inducing islet *β* cell apoptosis; however, whether these events are associated with the inhibition of the Akt remains unclear [10, 26, 27].

Our findings suggest the following: first, diabetes induces Par-4 and inhibits TERT expression and decreases their interaction, promoting islet *β* cell apoptosis, whereas inhibition of Par-4 relieves apoptosis, as previous studies have indicated. Our previous research revealed that ER stress induced by diabetes induces Par-4 expression in response to apoptotic signalling factors, which then activates the cell membrane and mitochondrial pathways to induce the apoptosis of islet *β* cells. Thus, Par-4 is important for induction of apoptosis in diabetes, but what regulates Par-4 expression is still under investigation. The present study indicated this process involves the interaction of TERT with Par-4 in the cytoplasm, which then translocates to induce islet *β* cell apoptosis. After high-glucose/fatty acid intervention, Par-4 expression levels and apoptosis increase; cytoplasmic TERT levels decrease, interaction between Par-4 and TERT occurs in the cytoplasm, and cell survival and insulin secretion were decreased. After inhibition of Par-4, cytoplasmic TERT levels increase, the interaction between Par-4 and TERT in the cytoplasm increases, apoptosis decreases, and cell survival and insulin secretion increase. Second, Par-4 may participate in islet *β* cell apoptosis and dysfunction induced by high-glucose/fatty acids through the Akt signalling pathway. The Akt signalling pathway governs multiple biological processes through the regulation of various target genes. However, inhibition of Akt signalling or genetic mutations in the Akt signalling pathway often lead to loss of proliferation and, thus, to apoptosis. Par-4 is an important upstream target of the Akt signalling pathway in the regulation of apoptosis, but whether there are any additional factors associated with Akt and Par-4 in diabetic islet *β* cell apoptosis is still unclear. Our study provides the first evidence that Par-4 can inhibit the phosphorylation of Akt to induce islet *β* cell apoptosis. Diabetes promotes the secretion and expression of Par-4, increases nuclear Par-4 levels in islet *β* cells, and inhibits Akt phosphorylation, consequently promoting islet *β* cell apoptosis and reducing islet *β* cell survival and glucose-stimulated insulin secretion. Inhibiting the expression of Par-4 suppresses these processes. Moreover, treatment with a p-Akt inhibitor in a diabetes model aggravates apoptosis, cell survival, and islet *β* cell dysfunction, and inhibiting Par-4 expression relieves these processes. Third, we found that TERT (170–546 aa) can bind to the NLS and leucine zipper domains of Par-4.

Par-4 is a well-established tumour suppressor, based on the fact that it can induce apoptosis in various types of tumour cells. Our data demonstrating a novel role of Par-4 in diabetes suggest that Par-4 could also function as a diabetes promoter due to its apoptosis induction effect on islet *β* cells. The possible mechanism whereby Par-4 mediates islet *β* cell apoptosis in diabetes likely involves an association with the TERT and Akt signalling pathway. We first revealed that the binding of TERT and Par-4 is part of a pathway that is independent of the typical telomere-telomerase system and participates in the regulation of islet cell apoptosis. Par-4 has previously been shown to physically interact with many factors. In the present study, we showed that under normal conditions, Par-4 and TERT bind with each other and have no effect on apoptosis, and Par-4 was found to accumulate in nuclear extracts following inhibition of TERT and high-glucose/fatty acid treatment. These findings suggest that diabetes and TERT inhibition can drive Par-4 translocation to the nucleus to induce islet *β* cell apoptosis. The increase in Par-4 translocation to the nucleus also inhibits Akt phosphorylation to induce islet *β* cell apoptosis and dysfunction. This work elucidates the specific mechanism of binding between TERT and Par-4 that is involved in islet *β* cell apoptosis and provides a novel target for gene therapy in type 2 diabetes and other age-related diseases.

## Data Availability

The data used to support the findings of this study are available from the corresponding author upon request.

## Supplementary Materials

Supplementary Materials The supplementary materials contain supplementary data: report for nuclease microinjection product of Par-4 knockout mouse and Supplementary Tables 1–10.
