# Corrigendum to “TERT and Akt Are Involved in the Par-4-Dependent Apoptosis of Islet *β* Cells in Type 2 Diabetes”

**DOI:** 10.1155/2020/1912058

**Published:** 2020-01-22

**Authors:** Chen Liu, Wu QiNan, Lei XiaoTian, Yang MengLiu, Gan XiaGuang, Leng WeiLing, Liang ZiWen, Zhang Ling, Yang GangYi, Chen Bing

**Affiliations:** ^1^Endocrine Department, First Affiliated Hospital of the Third Military Medical University (Army Medical University), Chongqing 400038, China; ^2^Endocrine Department, Second Affiliated Hospital of Chongqing Medical University, Chongqing 400010, China; ^3^Outpatient Department, First Affiliated Hospital of the Third Military Medical University (Army Medical University), Chongqing 400038, China

In the article titled “TERT and Akt Are Involved in the Par-4-Dependent Apoptosis of Islet *β* Cells in Type 2 Diabetes” [1], the image of Figure 3(d) that represents the TERT expression of group C was incorrect. Also, the western blot signs of TERT and GADPH in Figure 6(c) were swapped. The authors stated that the original figures of the article are not available. The correct figures are as follows:

## Figures and Tables

**Figure 1:**
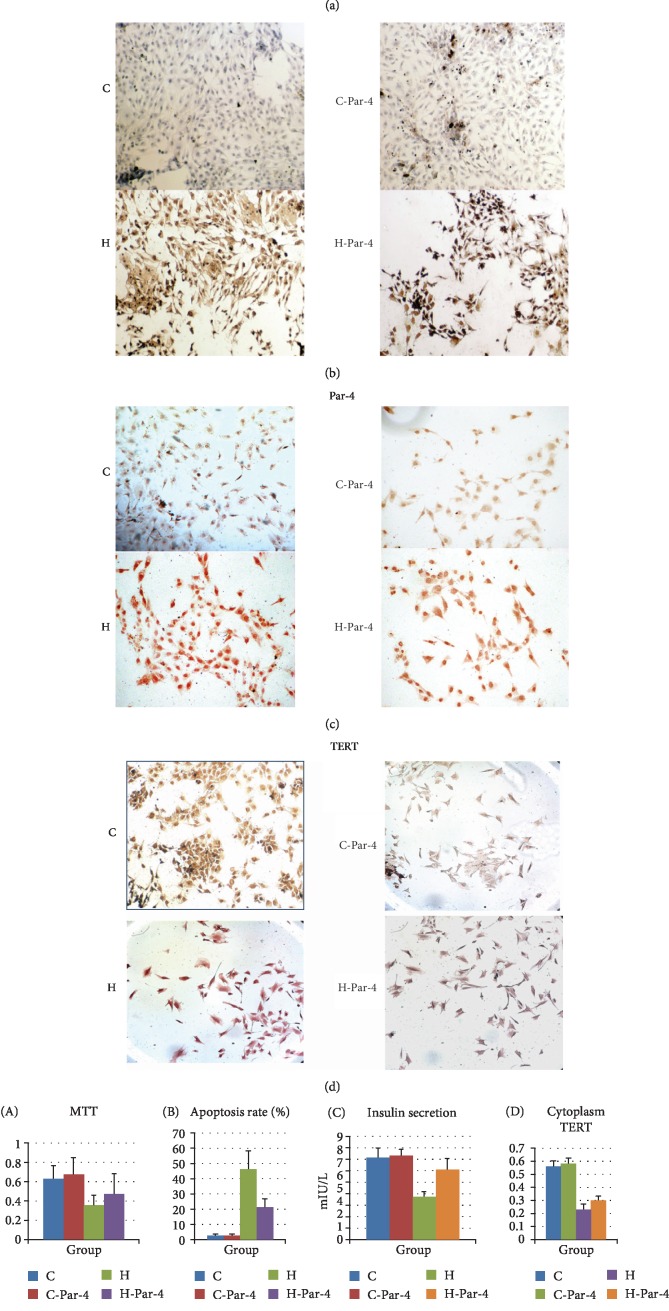
Subcellular localization of Par-4 and TERT in diabetes. (a) Western blot analysis of Par-4 and TERT expression in cytoplasmic and nuclear extracts in each group. (b) Apoptosis was detected via TUNEL staining in each group. (c) Immunocytochemistry analysis of Par-4 expression in each group. (d) Immunocytochemistry analysis of TERT expression in each group. (e) Expression of Par-4 and TERT, apoptosis rates, cell survival rates determined with MTT assays, and glucose-stimulated insulin secretion in each group. Compared with the C group (*P* < 0.05). ^#^Compared with the H group (*P* < 0.05).

**Figure 2:**
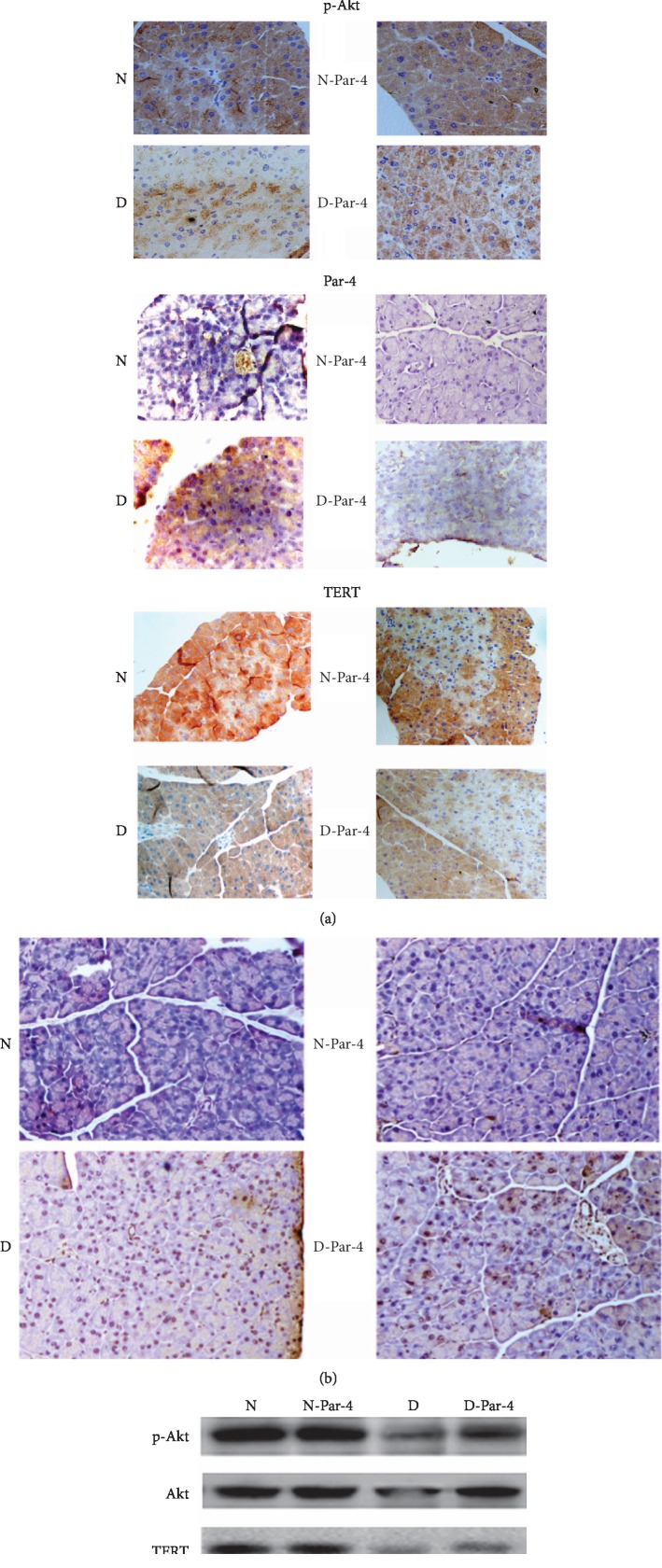
Diabetes activates Par-4 and inhibits TERT and p-Akt, inducing islet *β* cell apoptosis. (a) Immunocytochemistry analysis of Par-4 and TERT expression in cytoplasmic and nuclear extracts in each group. (b) Apoptosis was detected via TUNEL staining in each group. (c) Western blots of Par-4, TERT, Akt, and p-Akt expression in each group. (d) Expression of Par-4 (A), TERT (B), Akt (C), p-Akt (D), apoptosis rates (E), secretion of Par-4 (F), the HOMA-*β* index (G), and glucose-stimulated insulin secretion (H) in each group. (e) Signal transduction via the Par-4/TERT-Akt pathway to induce islet *β* cell apoptosis in diabetes. Compared with the N group (*P* < 0.05). ^#^Compared with the D group (*P* < 0.05).

## References

[1] Liu C., QiNan W., XiaoTian L. (2018). TERT and Akt are involved in the Par-4-dependent apoptosis of islet *β* cells in type 2 diabetes. *Journal of Diabetes Research*.

