# Supplementary material for: TERT and Akt Are Involved in the Par-4-Dependent Apoptosis of Islet β Cells in Type 2 Diabetes
Source: J Diabetes Res. 2018 Aug 14;2018:7653904. doi: 10.1155/2018/7653904 (PMC6112224; doi:10.1155/2018/7653904)
Supplement: Supplementary Materials — The supplementary materials contain supplementary data: report for nuclease microinjection product of Par-4 knockout mouse and Supplementary Tables 1–10. [file 7653904.f1.doc]

**Supplement data**

**Report for Nuclease Microinjection Product**

| Customer |  | | | |  |
| --- | --- | --- | --- | --- | --- |
| Contract No. | KOTS131118BA1 | | | |  |
| Report Date | 2015-11-18 | | | |  |
| **Project Summary** | | | | |  |
| **Objective:** To create mPawr knockout in C57BL/6 mice by microinjection of TALENs in fertilized eggs.  **Summary:**   - The mPawr gene (GenBank accession number: NM_054056.2; Ensembl: ENSMUSG00000035873) is located on mouse chromosome 10. - Exon 2 was selected as target site. - TALEN mRNA generated by *in vitro* transcription was then injected into fertilized eggs for KO mouse productions. - The founders were genotyped by PCR followed by DNA sequencing analysis. - The positive founders were breeding to the next generation which was genotyped by PCR and DNA sequencing analysis. | | | | |  |
| **Results** | | | | |  |
| Name of Injected mRNA | mPawr-2-L/R | | | |  |
| Mouse Strain | C57BL/6 | | | |  |
| Date of Birth | 2015-04-06 | | | |  |
| Founders (F0) Generated | ♂ | 1 | | Mouse-ID#1 |  |
|  | ♀ | 1 | | Mouse-ID#4 |  |
| Mouse Strain | C57BL/6(Mouse-ID#1) × C57BL/6 (WT) | | | |  |
| Date of Birth | 2015-06-13 | | | |  |
| Founders (F1) Generated | ♂ | 2 | | Mouse-ID#5, Mouse-ID#7 |  |
|  | ♀ | 2 | | Mouse-ID#1, Mouse-ID#2 |  |
| Mouse Strain | C57BL/6(Mouse-ID#4) × C57BL/6 (WT) | | | |  |
| Date of Birth | 2015-06-26 | | | |  |
| Founders (F1) Generated | ♂ | 2 | | Mouse-ID#22, Mouse-ID#24 |  |
| 1. **The 249 bp long products were generated from PCR genotyping using the primers and conditions listed below. The amplicons were then purified and sent for DNA sequencing analysis.**  - mPawr-F: 5’-CACCTTTGTCCAGAAGGTTAGCAGA-3’ - mPawr-R: 5’-CTGGTCACTTTGAACGCTCACCT-3’ - Product Size: 249 bp - Annealing Temp: 59℃  1. **DNA sequencing using the primer listed below revealed that Mouse-ID#1 was missing 11 bases in one strand and 12 bases in the other strand; Mouse-ID#4 was missing 1 base in one strand. Please note that wildtype DNA was used as a negative control for sequencing in parallel. In this way a direct comparison can be made with DNA of similar quality, and used as a baseline to help identify minor peaks. Each mutant mouse’s chromatogram (Fig. 1, 2, 3, 4 and 5) indicated lower sequence quality starting at the specified locus. This is typical as the deletion leads to mixed sequence population and confuses sequencing software’s ability to call out given bases.**  - DNA Sequencing Primer (Forward Sequencing): - 5’-CACCTTTGTCCAGAAGGTTAGCAGA-3’   Pairwise Sequence Comparison  Wildtype 5’-tgttctttctctcagtgcttagatgagtacgaagatgatgaagcaggacagaaggaacggaa-3’  ID#1 5’-tgttctttctctcagtgcttagatgagt-----------gaagcaggacagaaggaacggaa-3’ (-11)  ID#1’ 5’-tgttctttctctcagtgcttagatga------------tgaagcaggacagaaggaacggaa-3’ (-12)  ID#4 5’-tgttctttctctcagtgcttagatgag-acgaagatgatgaagcaggacagaaggaacggaa-3’ (-1)  DNA Sequence Chromatograms   1. Mouse-ID#1:11 bases deletion (ACGAAGATGAT) in one strand.   ID#1 5’-tgttctttctctcagtgcttagatgagt-----------gaagcaggacagaaggaacggaa-3’ (-11)  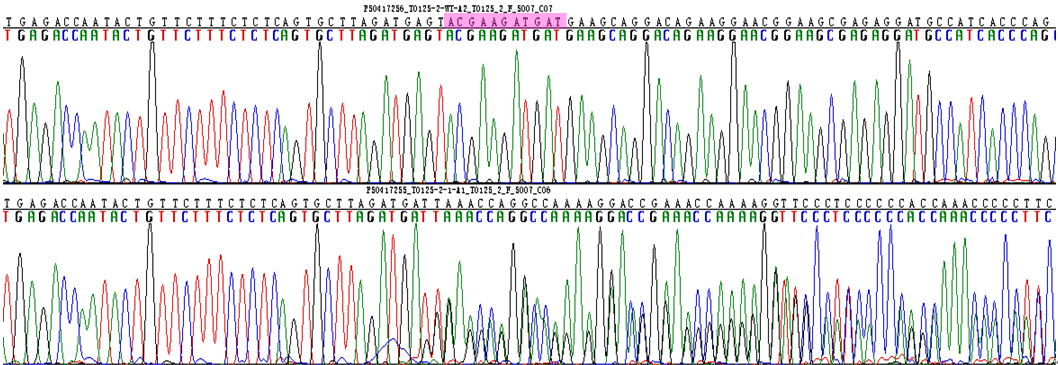  Fig.1. (Top) DNA sequences of wildtype mouse. (Bottom) DNA sequences of Mouse-ID#1.  ID#1-F1-Mouse-ID#1, #2, #5, #7: 11 bases deletion (ACGAAGATGAT) in one strand.  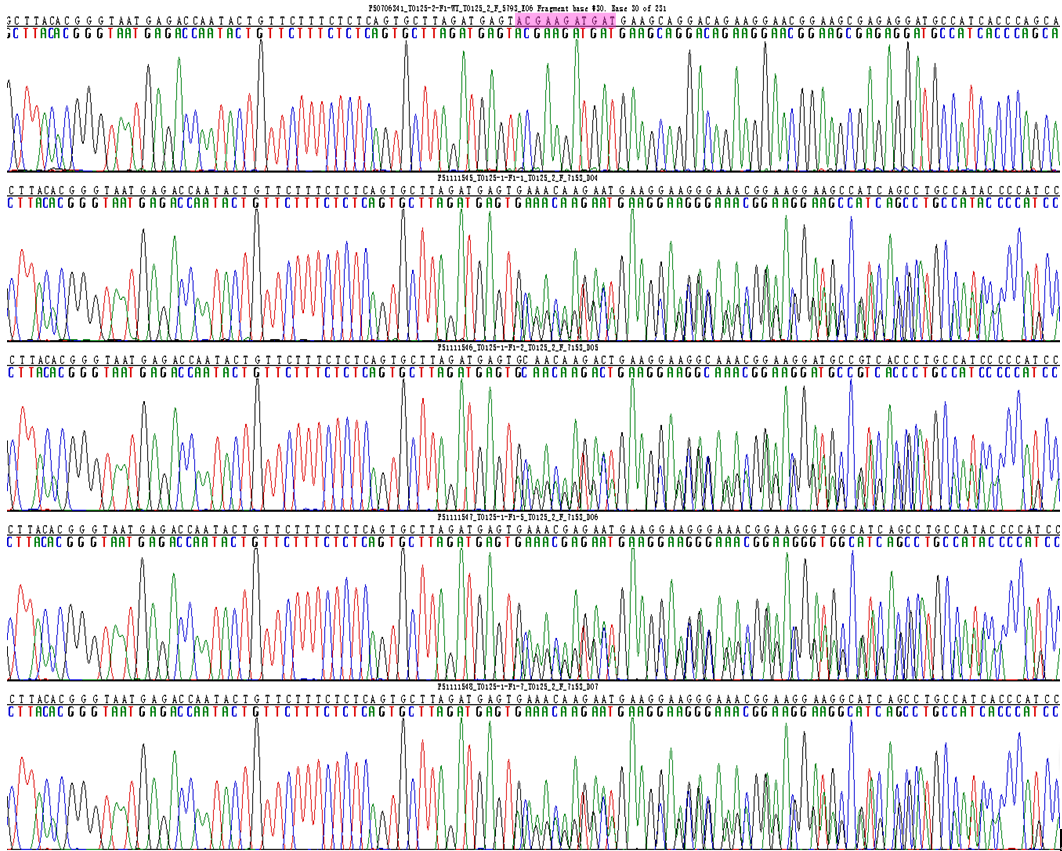  Fig.2. (Top) DNA sequences of wildtype mouse. (Bottom) DNA sequences of ID#1-F1-Mouse-ID#1, #2, #5, #7.   1. Mouse-ID#1’: 12 bases deletion (GTACGAAGATGA).   ID#1’ 5’-tgttctttctctcagtgcttagatga------------tgaagcaggacagaaggaacggaa-3’ (-12)  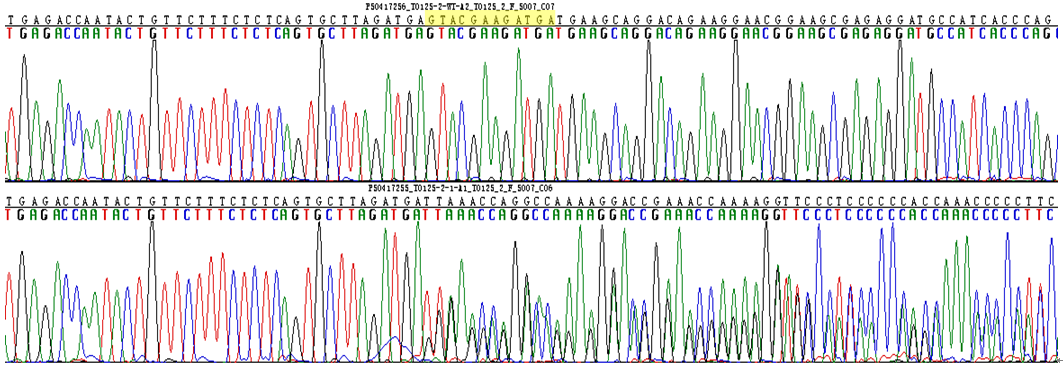  Fig.3. (Top) DNA sequences of wildtype mouse. (Bottom) DNA sequences of Mouse-ID#1’.   1. Mouse-ID#4: 1 base deletion (T) in one strand.   ID#4 5’-tgttctttctctcagtgcttagatgag-acgaagatgatgaagcaggacagaaggaacggaa-3’ (-1)  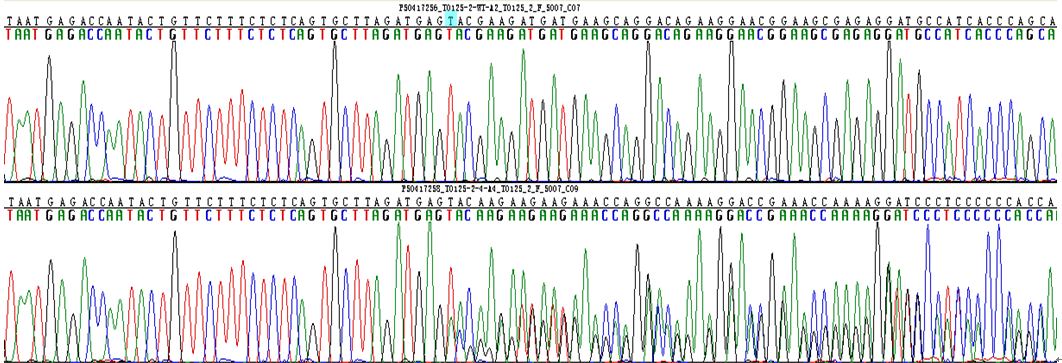  Fig.4. (Top) DNA sequences of wildtype mouse. (Bottom) DNA sequences of Mouse-ID#4.  ID#4-F1-Mouse-ID#22, #24: 1 base deletion (T) in one strand.  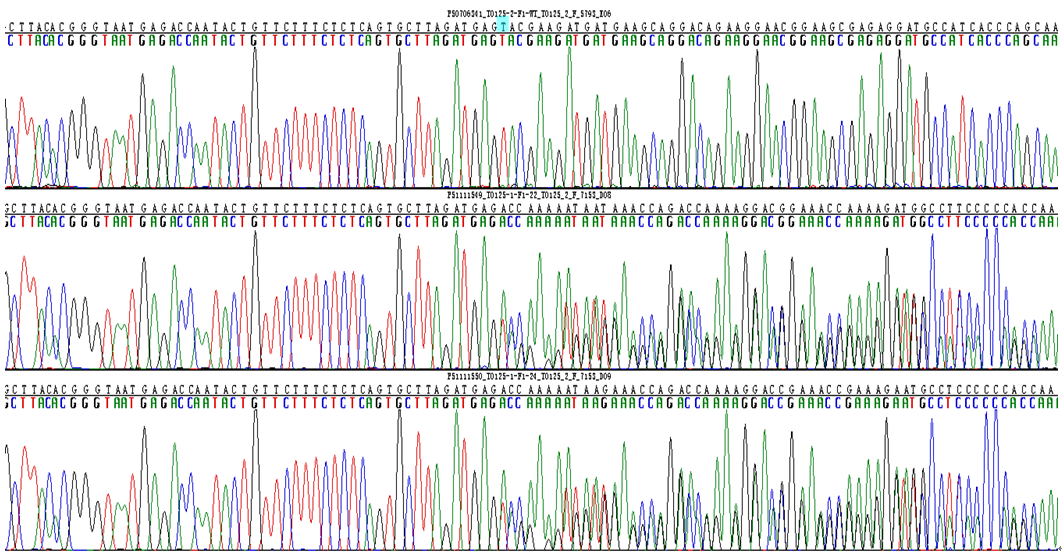  Fig.5. (Top) DNA sequences of wildtype mouse. (Bottom) DNA sequences of ID#1-F1-Mouse-ID#22, #24.   1. **The mRNA transcribed from targeted allele with frameshift undergoes nonsense- mediated decay (NMD).**   ID#1-F1-Mouse-ID#1, #2, #5, #7:  1 MATGGYRSGG STTTDFLEEW KAKREKMRAK QNPAGPGSSG GDPAAKSPAG  51 SLTPTAVAGT SELNHGPAGA AAPAAPAPGA LNCAHGSSTL PRAAPGSRRA  101 EDECPSAAAA SGAPGSRGDE EEPDSAREKG RSSGPSARKG KGQIEKRKLR  151 EKRRSTGVVN IPAAECLDE  ID#4-F1-Mouse-ID#22, #24:  1 MATGGYRSGG STTTDFLEEW KAKREKMRAK QNPAGPGSSG GDPAAKSPAG  51 SLTPTAVAGT SELNHGPAGA AAPAAPAPGA LNCAHGSSTL PRAAPGSRRA  101 EDECPSAAAA SGAPGSRGDE EEPDSAREKG RSSGPSARKG KGQIEKRKLR  151 EKRRSTGVVN IPAAECLDEY KMMKQDRRNG SERMPSPSKT PSRMKLRPSQ  201 IQAHPTCPRT RREQFQADTK AQPVPQKMKS QIDIPEQTEV VSVDTTEMQM  251 RRLVSPQVAP WKRELKILKR KL | | | | |  |
| **Relevant Reagents** | | | | | |
| Water | | | Sigma, Cat. No. W1503 | | |
| EDTA | | | Sigma, Cat. No. E7889 | | |
| Trizma Hydrochloride Solution | | | Sigma, Cat. No. T2663 | | |
| Proteinase K | | | Merck, Cat. No. MK539480 | | |
| Taq DNA Polymerase | | | Takara, Cat. No. R007 | | |
| dNTP | | | Takara, Cat. No. 4030 | | |
| Agarose | | | Biowest Agarose, Regular | | |
| DNA Marker | | | FermentasGeneRuler™ 100bp DNA Ladder #[SM0241](http://www.fermentas.com/profiles/electrophoresis/pdf/generuler0241_impr.pdf) | | |
| 0.5×TBE | | | Tris Bio Basic Inc, TBO194-500g  EDTA Shanghai Sangon, 0105-500g  Boric Acid, Shanghai Sangon, 0588-500g | | |

**Supplement Table**

Table 1 FBG, HbA1c, FINS, C-peptide, and HOMA-β in the type 2 diabetes and healthy groups

| Group | FBG  (mmol/L) | HbA1c  (mmol/L) | FINS  (mIU/L) | C-peptide  (nmol/L) | HOMA-β  (%) |
| --- | --- | --- | --- | --- | --- |
| Control | 5.34±0.48 | 4.81±0.73 | 13.64±3.44 | 1.58±0.64 | 160.12±68.0 |
| Diabetes | 9.16±3.22* | 7.67±1.90* | 12.88±8.85 | 1.29±0.41* | 48.9±27.08* |

* Compared with the control group, p<0.05

Table 2 Par-4, TERT, TC, TG, LDL-C and HDL-C in the type 2 diabetes and healthy groups

|  | Par-4 (ng/ml) | TERT  (ng/ml) | TC  (mmol/L) | TG  (mmol/L) | LDL-C  (mmol/L) | HDL-C  (mmol/L) |
| --- | --- | --- | --- | --- | --- | --- |
| Control | 14.08±2.94 | 80.29±5.11 | 5.50±1.73 | 1.56±0.86 | 3.59±1.35 | 1.61±0.45 |
| Diabetes | 17.44±3.65* | 48.56±7.80* | 6.20±1.65 | 3.73±3.44* | 3.62±1.30 | 1.23±0.40* |

* Compared with the control group, p<0.05

| Table 3 The correlations among the measured parameters in the type 2 diabetes and healthy groups | | | | | | | | | | | | |
| --- | --- | --- | --- | --- | --- | --- | --- | --- | --- | --- | --- | --- |
|  | | | Insulin | C-peptide | HOMA-β | Par-4 | TG | TC | LDL-C | HDL-C | HbA1c | TERT |
| Spearman ‘s  r ho | Insulin | Correlation  Index | 1.000 | .168 | .486 | -.251 | -.026 | -.162 | .184 | -.013 | -.079 | .161 |
|  |  | Sig. (double) | . | .199 | .000 | .053 | .841 | .217 | .160 | .923 | .546 | .220 |
|  |  | N | 60 | 60 | 60 | 60 | 60 | 60 | 60 | 60 | 60 | 60 |
|  | C-peptide | Correlation  Index | .168 | 1.000 | .224 | -.337 | -.044 | -.040 | -.176 | -.091 | -.195 | .194 |
|  |  | Sig. (double) | .199 | . | .085 | .008 | .740 | .759 | .179 | .488 | .135 | .138 |
|  |  | N | 60 | 60 | 60 | 60 | 60 | 60 | 60 | 60 | 60 | 60 |
|  | HOMA-β | Correlation  Index | .486 | .224 | 1.000 | -.365 | -.306 | -.441 | .364 | -.178 | -.736 | .716 |
|  |  | Sig. (double) | .000 | .085 | . | .004 | .017 | .000 | .004 | .174 | .000 | .000 |
|  |  | N | 60 | 60 | 60 | 60 | 60 | 60 | 60 | 60 | 60 | 60 |
|  | Par-4 | Correlation  Index | -.251 | -.337 | -.365 | 1.000 | .197 | .081 | -.117 | .125 | .270 | -.362 |
|  |  | Sig. (double) | .053 | .008 | .004 | . | .132 | .538 | .372 | .343 | .037 | .004 |
|  |  | N | 60 | 60 | 60 | 60 | 60 | 60 | 60 | 60 | 60 | 60 |
|  | TG | Correlation  Index | -.026 | -.044 | -.306 | .197 | 1.000 | .397 | .084 | .710 | .434 | -.278 |
|  |  | Sig. (double) | .841 | .740 | .017 | .132 | . | .002 | .523 | .000 | .001 | .032 |
|  |  | N | 60 | 60 | 60 | 60 | 60 | 60 | 60 | 60 | 60 | 60 |
|  | TC | Correlation  Index | -.162 | -.040 | -.441 | .081 | .397 | 1.000 | -.524 | .213 | .505 | -.387 |
|  |  | Sig. (double) | .217 | .759 | .000 | .538 | .002 | . | .000 | .102 | .000 | .002 |
|  |  | N | 60 | 60 | 60 | 60 | 60 | 60 | 60 | 60 | 60 | 60 |
|  | LDL-C | Correlation  Index | .184 | -.176 | .364 | -.117 | .084 | -.524 | 1.000 | .088 | -.359 | .314 |
|  |  | Sig. (double) | .160 | .179 | .004 | .372 | .523 | .000 | . | .503 | .005 | .015 |
|  |  | N | 60 | 60 | 60 | 60 | 60 | 60 | 60 | 60 | 60 | 60 |
|  | HDL-C | Correlation  Index | -.013 | -.091 | -.178 | .125 | .710 | .213 | .088 | 1.000 | .203 | -.125 |
|  |  | Sig. (double) | .923 | .488 | .174 | .343 | .000 | .102 | .503 | . | .120 | .341 |
|  |  | N | 60 | 60 | 60 | 60 | 60 | 60 | 60 | 60 | 60 | 60 |
|  | HbA1c | Correlation  Index | -.079 | -.195 | -.736 | .270 | .434 | .505 | -.359 | .203 | 1.000 | -.742 |
|  |  | Sig. (double) | .546 | .135 | .000 | .037 | .001 | .000 | .005 | .120 | . | .000 |
|  |  | N | 60 | 60 | 60 | 60 | 60 | 60 | 60 | 60 | 60 | 60 |
|  | TERT | Correlation  Index | .161 | .194 | .716 | -.362 | -.278 | -.387 | .314 | -.125 | -.742 | 1.000 |
|  |  | Sig. (double) | .220 | .138 | .000 | .004 | .032 | .002 | .015 | .341 | .000 | . |
|  |  | N | 60 | 60 | 60 | 60 | 60 | 60 | 60 | 60 | 60 | 60 |

Table 4 The apoptosis rate, Par-4 expression, TERT expression, MTT results and insulin secretion in each group**($\overline{X}$±s)**

| Group | Apoptosis  Rate (%) | Par-4 | TERT | MTT  OD450 | Insulin  Secretion |
| --- | --- | --- | --- | --- | --- |
| C  H12  H24  H48 | 2.63±0.92#  10.50±2.20*#  25.13±3.00*  46.63±11.94*# | 0.30±0.07#  0.45±0.06*#  0.73±0.12*  0.92±0.04*# | 0.71±0.09#  0.53±0.02*  0.37±0.12*  0.36±0.05# | 0.64±0.13#  0.58±0.13*#  0.47±0.09*  0.36±0.10*# | 7.17±0.87#  5.66±0.60*#  4.54±0.60*  3.76±0.40*# |

* Compared with the C group, p<0.05

# Compared with the H24 group, p<0.05

Table 5 The apoptosis rate, cytoplasmic and nuclear Par-4 and TERT expression, MTT and insulin secretion in each group($\overline{X}$**±s**)

| Group | Apoptosis  Rate (%) | Cytoplasm  Par-4 | Cytoplasm  TERT | Nuclear  Par-4 | Nuclear  TERT | MTT  OD450 | Insulin  Secretion |
| --- | --- | --- | --- | --- | --- | --- | --- |
| C  C-Par-4  H  H-Par-4 | 2.63±0.92#  2.88±0.83#  46.63±11.94*  21.63±5.71*# | 0.60±0.06#  0.25±0.04*#  1.22±0.06*  0.72±0.08*# | 0.56±0.04#  0.58±0.04  0.23±0.04*  0.30±0.03# | 0.64±0.07#  0.29±0.02*#  1.77±0.07*  1.12±0.11*# | 0.50±0.05  0.52±0.04  0.53±0.06  0.51±0.03 | 0.64±0.13#  0.68±0.17#  0.36±0.10*  0.47±0.22*# | 7.17±0.87#  7.32±0.63#  3.76±0.40*  6.10±1.01*# |

* Compared with the C group, p<0.05

# Compared with the H group, p<0.05

**Table 6 Apoptosis rate, insulin secretion, MTT results, and Par-4, Akt and p-Akt expression in each group (($\overline{X}$±s))**

| Group | Apoptosis  Rate (%) | Par-4 | Akt | p-Akt | MTT  OD450 | Insulin  Secretion |
| --- | --- | --- | --- | --- | --- | --- |
| C  CS  H  HS | 2.63±0.92#  2.88±0.99#  46.63±11.94*  66.00±14.36*# | 0.45±0.07#  0.44±0.03#  1.78±0.08*  2.39±0.09*# | 1.61±0.19#  1.63±0.08#  0.46±0.12*  0.49±0.10*# | 2.24±0.22#  1.31±0.09#  0.84±0.14*  0.58±0.05*# | 0.64±0.13#  0.48±0.10#  0.36±0.10*  0.22±0.10*# | 7.17±0.87#  4.82±0.91#  3.76±0.40*  2.12±0.22*# |

* Compared with the C group, p<0.05

# Compared with the H group, p<0.05

**Table 7 Apoptosis rate, insulin secretion, MTT results, and Par-4, Akt and p-Akt expression in each group (($\overline{X}$±s))**

| Group | Apoptosis  Rate (%) | Par-4 | Akt | p-Akt | MTT  OD450 | Insulin  Secretion |
| --- | --- | --- | --- | --- | --- | --- |
| CS  HS  CS-Par-4  HS-Par-4 | 2.88±0.83#  66.00±14.36*  2.25±0.89#  14.13±3.64*# | 0.84±0.05#  2.50±0.11*  0.43±0.09#  1.31±0.06*# | 0.84±0.05#  0.63±0.09*  0.81±0.10#  0.65±0.09* | 0.99±0.05#  0.41±0.04*  0.99±0.03#  0.80±0.11*# | 0.48±0.10#  0.22±0.10*  0.39±0.09#  0.31±0.06*# | 4.82±0.91#  2.12±0.22*  5.06±0.93#  3.77±0.59*# |

* Compared with the CS group, p<0.05

# Compared with the HS group, p<0.05

**Table 8 Weight, HOMA-β, apoptosis rate, insulin secretion, and Par-4, TERT, Akt, and p-Akt expression in each group ($\overline{X}$±s)**

|  | N | N-Par-4 | D | D-Par-4 |
| --- | --- | --- | --- | --- |
| Weight (g)  Insulin (mIU/L)  HOMA-β  Apoptosis rate (%)  TERT  Par-4  Akt  p-Akt  Par-4 Secretion(ng/ml) | 20.35±0.75#  21.72±2.57#  361.01±211.84#  3.63±2.13#  1.48±0.08#  0.95±0.02#  2.47±0.16#  1.77±0.15#  62.56±12.23# | 21.70±0.82#  21.69±1.48#  302.73±153.14  3.75±1.98#  1.51±0.10#  0.03±0.01*#  2.57±0.11#  1.75±0.10#  4.33±2.76*# | 35.29±3.17*  14.71±1.50*  56.26±15.18#  44.75± 11.45*  0.66±0.09*  2.19±0.04*  0.62±0.07*  0.57±0.05*  397.38±82.86* | 35.70±3.60*  25.02±2.27*#  115.01±12.07*  22.5±6.68*#  0.86±0.07*#  0.03±0.01*#  0.91±0.09*#  0.96±0.03*#  4.64±3.24*# |

* Compared with the N group, P<0.05

# Compared with the D group, P<0.05

Table 9 Kinetic analysis of the interaction between Par-4 and TERT-2

| Ligand | Analyte | Con.(nM) | Response | K_on_(1/Ms) | K_off_(1/s) | K_D_(M) | Full Rˆ2 |
| --- | --- | --- | --- | --- | --- | --- | --- |
| Par-4 | TERT-2 | 840 | -0.9606 | 1.19E+04 | <1.0E-07 | <1.0E-12 | 0.2823 |
| Par-4 | TERT-2 | 646.2 | -0.4221 | 1.19E+04 | <1.0E-07 | <1.0E-12 | 0.2823 |
| Par-4 | TERT-2 | 497 | 0.2626 | 1.19E+04 | <1.0E-07 | <1.0E-12 | 0.2823 |
| Par-4 | TERT-2 | 382.3 | 0.5184 | 1.19E+04 | <1.0E-07 | <1.0E-12 | 0.2823 |
| Par-4 | TERT-2 | 294.1 | 0.0345 | 1.19E+04 | <1.0E-07 | <1.0E-12 | 0.2823 |

- Equilibrium dissociation constant K_D_: affinity strength
- Binding rate constant K_on_: rate of formation/unit time
- Dissociation rate constant K_dis_: percentage of degradation/unit time

Table 10 Kinetic analysis of the interaction between Par-4-1 and TERT-2(tag free)

| Ligand | Analyte | Con.  (nM) | Response | K_on_(1/Ms) | K_off_(1/s) | K_D_(M) | Full Rˆ2 |
| --- | --- | --- | --- | --- | --- | --- | --- |
| Par-4-1 | TERT-2(tag free) | 37.5 | 0.4927 | 8.23E+04 | 1.49E-04 | 1.81E-09 | 0.9949 |
| Par-4-1 | TERT-2(tag free) | 18.8 | 0.1692 | 8.23E+04 | 1.49E-04 | 1.81E-09 | 0.9949 |
| Par-4-1 | TERT-2(tag free) | 9.38 | 0.0705 | 8.23E+04 | 1.49E-04 | 1.81E-09 | 0.9949 |
| Par-4-1 | TERT-2(tag free) | 4.69 | 0.0506 | 8.23E+04 | 1.49E-04 | 1.81E-09 | 0.9949 |
| Par-4-1 | TERT-2(tag free) | 2.34 | 0.029 | 8.23E+04 | 1.49E-04 | 1.81E-09 | 0.9949 |
| Par-4-2 | TERT-2(tag free) | 37.5 | 0.3037 | 2.20E+04 | <1.0E-07 | <1.0E-12 | 0.9466 |
| Par-4-2 | TERT-2(tag free) | 18.8 | 0.0888 | 2.20E+04 | <1.0E-07 | <1.0E-12 | 0.9466 |
| Par-4-2 | TERT-2(tag free) | 9.38 | 0.0352 | 2.20E+04 | <1.0E-07 | <1.0E-12 | 0.9466 |
| Par-4-2 | TERT-2(tag free) | 4.69 | 0.0445 | 2.20E+04 | <1.0E-07 | <1.0E-12 | 0.9466 |
| Par-4-2 | TERT-2(tag free) | 2.34 | 0.0329 | 2.20E+04 | <1.0E-07 | <1.0E-12 | 0.9466 |
| Par-4-3 | TERT-2(tag free) | 37.5 | 0.4329 | 7.90E+04 | 2.04E-07 | 2.58E-12 | 0.9493 |
| Par-4-3 | TERT-2(tag free) | 18.8 | 0.0719 | 7.90E+04 | 2.04E-07 | 2.58E-12 | 0.9493 |
| Par-4-3 | TERT-2(tag free) | 9.38 | -0.0022 | 7.90E+04 | 2.04E-07 | 2.58E-12 | 0.9493 |
| Par-4-3 | TERT-2(tag free) | 4.69 | 0.0045 | 7.90E+04 | 2.04E-07 | 2.58E-12 | 0.9493 |
| Par-4-3 | TERT-2(tag free) | 2.34 | 0.0147 | 7.90E+04 | 2.04E-07 | 2.58E-12 | 0.9493 |

- Equilibrium dissociation constant K_D_: affinity strength
- Binding rate constant K_on_: rate of formation/unit time
- Dissociation rate constant K_dis_: percentage of degradation/unit time
